# Isolation of Inositol Hexaphosphate (IHP)-Degrading Bacteria from Arbuscular Mycorrhizal Fungal Hyphal Compartments Using a Modified Baiting Method Involving Alginate Beads Containing IHP

**DOI:** 10.1264/jsme2.ME15206

**Published:** 2016-07-05

**Authors:** Shintaro Hara, Masanori Saito

**Affiliations:** 1 Graduate School of Agricultural Science, Tohoku University232–3 Yomogida, Naruko-onsen, Osaki, Miyagi 989–6711Japan

**Keywords:** phytate, AMF, alginate beads, *Sphingomonas* sp., *Caulobacter* sp

## Abstract

Phytate (inositol hexaphosphate; IHP)-degrading microbes have been suggested to contribute to arbuscular mycorrhizal fungi (AMF)-mediated P transfer from IHP to plants; however, no IHP degrader involved in AMF-mediated P transfer has been isolated to date. We herein report the isolation of IHP-degrading bacteria using a modified baiting method. We applied alginate beads as carriers of IHP powder, and used them as recoverable IHP in the AM fungal compartment of plant cultivation experiments. P transfer from IHP in alginate beads via AMF was confirmed, and extracted DNA from alginate beads was analyzed by denaturing gradient gel electrophoresis targeting the 16S rRNA gene and a clone library method for the beta-propeller phytase (BPP) gene. The diversities of the 16S rRNA and BPP genes of microbes growing on IHP beads were simple and those of *Sphingomonas* spp. and *Caulobacter* spp. dominated. A total of 187 IHP-utilizing bacteria were isolated and identified, and they were consistent with the results of DNA analysis. Furthermore, some isolated *Sphingomonas* spp. and *Caulobacter* sp. showed IHP-degrading activity. Therefore, we successfully isolated dominant IHP-degrading bacteria from IHP in an AMF hyphal compartment. These strains may contribute to P transfer from IHP via AMF.

Organic phosphorus constitutes a large proportion of soil P. The mineralization of organic P is one of the potentially important processes that regulate the availability of soil P to plants. *myo*-Inositol hexakisphosphate (IHP or phytate), which is derived in soil from plant residues, is the predominant form of organic P in soil (36). IHP is synthesized in plants and accumulates in seeds, in which it accounts for up to 90% of total organic P (36), and also occurs at low concentrations in leaves, roots, and other parts of plants (1). IHP is hydrolyzed by diverse microorganisms in soil (17, 20, 26, 37). However, IHP in soil forms insoluble mineral complexes with Ca, Fe, and Al (36); thus, it may not be readily decomposable. It is not yet well understood how IHP in soil is mineralized and becomes available to plants (33).

Arbuscular mycorrhizal fungi (AMF) are plant root symbionts that increase plant growth. AMF extend their hyphae to distant and large volumes of soil in order to acquire P, N, Zn, and other nutrients, which they transport to host plants. AMF acquire only the inorganic form of P (38). In other words, AMF cannot directly obtain P from IHP by themselves because they lack the phytase gene (35), which encodes the enzyme that catalyzes the hydrolysis of IHP. However, it has been suggested that the co-existence of IHP-degrading microbes with AMF may enable the utilization of IHP (39). Previous studies demonstrated that AMF absorbs P from IHP with possible assistance from IHP-degrading microbes that release inorganic P from IHP (7, 39). However, direct evidence to show that IHP-degrading bacteria were involved in the increase observed in P uptake via AMF was not provided. Zhang *et al.* (41) examined the effects of the co-inoculation of AMF, *Rhizophagus irregularis*, and the IHP-degrading bacterium *Pseudomonas alcaligenes*, with the application of IHP, on P absorption by plants from IHP via AMF. They showed that the inoculation of IHP-degrading bacteria did not enhance the transfer of P from IHP to host plants; however, the degradation of IHP and microbial absorption of P released were enhanced (41). These findings suggest that the co-existence of IHP-degrading bacteria and AMF promotes the hydrolysis of IHP, whereas the transfer of inorganic P does not occur from IHP to plants.

The isolation of IHP-degrading microbes, which supply inorganic P released from IHP, is a key step in elucidating the influence of the interaction between AMF and IHP-degrading microbes on P uptake from IHP. Therefore, we attempted to isolate dominant IHP-degrading microbes around IHP from which AMF acquires P. In order to isolate microbes meeting the above requirements, we applied a modified baiting method combined with cultivation experiments. In this baiting method, a specific substrate for target microbes is incubated with soil or other isolation sources to enrich the target microbes on the substrate, and the substrate is subsequently used for the isolation of microbes (3). Hence, the substrate of the bating method needs to be recoverable from soil. However, Ca-IHP and Fe-IHP, which are common forms of IHP in soil, are difficult to recover from soil because of the powdery state of these compounds. Therefore, we used alginate beads as carriers of these powdery compounds. Alginate beads are often used as carriers of chemicals and microbes (13), and are stable in soil for at least 3 or 4 months (25).

The objective of this study was to confirm whether IHP is used by plants via AMF with the help of IHP-degrading bacteria in soil and also to isolate the IHP-degrading bacteria responsible for supplying P from IHP to the hyphae of AMF. We performed 2 experiments using the experimental system with a nylon-meshed hyphal compartment containing the IHP source. In Experiment 1, the effective combination of AMF and the soil inoculum was investigated in terms of P transfer from IHP to the plant via AMF. In this experiment, we used organic matter rich in IHP and confirmed that plants acquired P from IHP via AMF. In Experiment 2, IHP-degrading bacteria were isolated from alginate beads containing IHP. In order to examine whether the isolated IHP-degrading bacteria are dominant *in situ*, bacterial diversity and the diversity of the beta-propeller phytase (BPP) gene, which is one of the major phytases of soil bacteria (20), around the IHP beads were also analyzed by denaturing gradient gel electrophoresis (DGGE) and a clone library method. We were able to identify the major IHP bacteria responsible for P transfer from IHP to plants.

## Materials and Methods

### Preparation of the P source

Ca-IHP and Fe-IHP were prepared from Na-IHP (phytic acid: inositol hexaphosphoric acid dodecasodium salt from rice, Sigma-Aldrich, St. Louis, MO, USA) according to the method of Jackman and Black (16). The amounts of contaminating inorganic P in the Ca-IHP and Fe-IHP preparations were 0.6% and 0.4% of total P, respectively. Alginate beads containing each P source were prepared as follows: 20 mL of a viscous suspension containing each P source, 40 g L^−1^ of sodium alginate, and 4 mM α-cyclodextrin were dropped into a stirred solution of 20 g L^−1^ CaCl_2_ (25). The P source in the viscous suspension was either 156.7 g L^−1^ Ca-IHP, 187.4 g L^−1^ Fe-IHP, 13.8 g L^−1^ CaHPO_4_·2H_2_O, or no P supply. The added viscous drops readily solidified in the CaCl_2_ solution. The alginate beads had a slim string shape with ϕ 2.0-mm round sections, and were cut into pellets of 2–5 mm in length (Fig. S1). Each bead was washed with distilled water several times, and surface water was removed using Kimwipes. Total P in each type of bead was measured after wet digestion with sulfuric acid and hydrogen peroxide. The amount of beads applied to the pot was adjusted in terms of the total P content. Alginate beads without a P source contained no P.

### Biological materials and growth conditions

Regarding the inocula of soil microorganisms, the surface soil of an upland field or that from a deciduous forest in the Kawatabi Field Science Center, Tohoku University, Osaki, Japan was used. Each soil was classified as a non-allophanic Andosol according to the USDA soil taxonomy; non-allophanic Andosol has a high phosphate-absorption coefficient. The physicochemical properties of arable soil were: pH (H_2_O), 5.5; total C, 77.3 g C kg^−1^ dry soil; total N, 0.7 g N kg^−1^ dry soil; available phosphate, 3.9 mg P kg^−1^ dry soil (Truog method), while those of forest soil were: pH (H_2_O), 4.0; total C, 123.5 g C kg^−1^ dry soil; total N, 1.1 g N kg^−1^ dry soil; available phosphate, 3.9 mg P kg^−1^ dry soil (Truog method).

The AM fungal strains used in this study were *R. irregularis* DAOM197198 and *Claroideoglomus etunicatum* isolated from arable soil (30). AMF were propagated on *Trifolium repens* and *Paspalum notatum*, and spores with mycelia were extracted by a wet sieving method and washed with reverse osmosis-purified water several times in order to reduce microbial contamination from the inoculum. Approximately 200 spores were inoculated in each plant.

*Lotus japonicus* (Gifu B-129) seeds were surface sterilized with hypochlorite and then germinated on moistened filter paper at 25°C. Seedlings were cultivated in a sterilized loamy soil-sand mixture with or without the inoculation of AMF. After 9 weeks, one young plant was transferred to a plastic pot containing 300 g of the sterilized loamy soil-sand mixture.

**Fig. 1:**
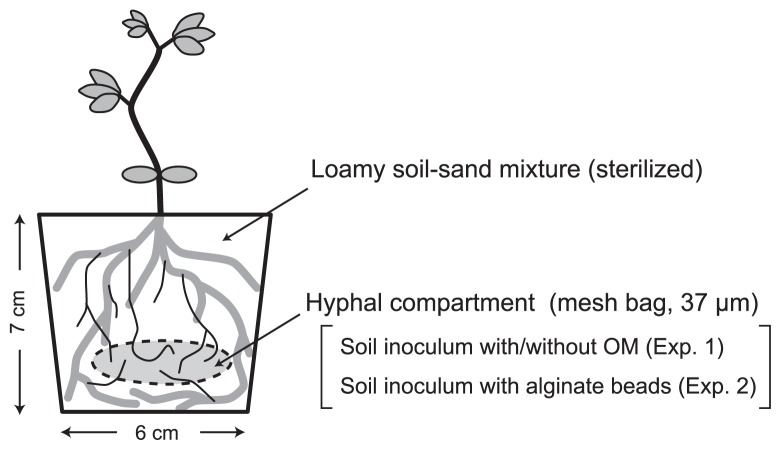
Schematic drawing of the experiment system. The hyphal compartment prevents the entry of plant roots, but not fungal hypha. The thin lines in the plastic pot indicate arbuscular mycorrhizal mycelia; the dotted line indicates a mesh bag made of a 37-μm nylon mesh.

A mesh bag (4 cm × 4 cm, 37-μm mesh), which contained 4.0 g dry weight of soil as the microorganism inoculum (arable soil or forest soil) with a P source or reference material, was placed under the plant in each pot. An outline of the experimental set-up is shown in Fig. 1.

Each experiment was established in a growth chamber. Growth conditions included a 16-h/8-h light/dark cycle at 22/20°C. Photosynthetically active radiation flux was 41.8 (± 2.8) μmol m^−2^ s^−1^ from the top, and 74.8 (± 21.4) μmol m^−2^ s^−1^ from the side. All compartments were watered daily with deionized water. Half-strength Hoagland nutrient solution containing 100 μM P (14) was applied once a week as follows: 10 mL for seedlings or 20 mL for the experimental phase.

### Experimental design

#### Experiment 1

Plants were cultivated with a combination of three mycorrhizal treatments (*R. irregularis*, *C. etunicatum*, or without an inoculation), two soil inocula in mesh bags (arable soil or forest soil), and two organic matter (OM) treatments (applied or not). In the IHP-rich OM application, a mixture of crushed soybeans and crushed buckwheat seeds (0.8 g pot^−1^, total P 4.25 mg P pot^−1^, phytate P 2.62 mg P pot^−1^, Truog P 0.10 mg P pot^−1^) was added to the mesh bag. In the “without OM” treatment, 1.2 mg of the superphosphate of lime (same quantity as OM in Truog P) was added to the mesh bag. The treatments were completely random with four replicates.

#### Experiment 2

Plants were cultivated with combinations of alginate beads containing several types of P sources in mesh bags and two mycorrhizal treatments (*R. irregularis* or without an inoculation). Alginate beads containing Ca-IHP, Fe-IHP, or CaHPO_4_ as a P source, or no P source was added to the mesh bag. The amount of IHP-containing beads was adjusted such that total P was equivalent to the amount of phytate P in 0.5 g of soybeans, and the amount of CaHPO_4_-containing beads was adjusted such that total P was equivalent to the amount of inorganic P in 0.5 g of soybeans. Phytate P in soybeans and buckwheat was measured as previously described (28). Arable soil was packed in mesh bags as soil inocula with alginate beads. The treatments were completely random with five replicates.

In both experiments, plants and samples were harvested 6 weeks after transplanting and used for analyses. Preliminary experiments showed that the percentage of the root length colonized taken by the intersection method (9) in AMF-inoculated treatments was more than 70% under these experimental conditions.

### Harvest and sample analysis

Plants were harvested after 6 weeks of growth with mesh bags. Alginate beads or the organic matter was immediately harvested from the mesh bag. Half of the alginate beads were used for bacterial isolation and the other half (or organic matter) were stored at −80°C for DNA extraction.

Shoots were oven dried at 90°C for 3 day and ground to a fine powder. Plant P concentrations were measured by the molybdenum blue reaction (23) after wet digestion in sulfuric acid and hydrogen peroxide.

### DNA extraction

DNA was extracted from the organic matter, alginate beads, and soil in mesh bags from the replicate pots using a commercial kit (ISOIL kit for Beads Beating, Nippon Gene, Tokyo, Japan), and pooled after extraction. Bead beating was performed using a Micro Smash MS-100 (Tomy Seiko, Tokyo, Japan) at 5,500 rpm for 45 s.

### DGGE analysis

A PCR-DGGE analysis was performed with KOD Fx Neo Polymerase (Toyobo, Osaka, Japan). The primer pair 341Fgc and 907R (2, 24) was used in the analysis of general bacteria, the primer pair alf19Fgc (5′-CGCCCGCCGCGCCCCGCGCCCGGCCCGCCGCCCCCGCCCC [GC clamp] CTGGCTCAGA(A/G)CGAACG-3′) and 518R (24) was used in the analysis of *Alphaproteobacteria*, and the primer pair NS1 and GCFund (22, 40) was used in the analysis of fungi. The *Alphaproteobacteria*-specific primer, alf19Fgc, was modified in this study from the probe used for fluorescence *in situ* hybridization (21).

The reaction mixture (50 μL) contained 1× KOD FX Neo buffer, 0.3 μM of each primer, 0.4 mM of each deoxynucleoside triphosphate, 1 U of KOD FX Neo polymerase, and template DNA (*ca.* 50 ng). In PCR of general bacteria and *Alphaproteobacteria*, DNA amplification was performed by touchdown PCR (6). During touchdown PCR, the annealing temperature, which was initially set at 65°C, was decreased by 0.5°C every cycle until a touchdown of 55°C, at which temperature 20 additional cycles were performed. In fungal PCR, the annealing temperature was 50°C and 30 reaction cycles were performed. The PCR program was as follows: an initial denaturing step at 94°C for 2 min followed by each cycle of denaturation at 98°C for 10 s, 30 s for primer annealing at the appropriate temperature, extension at 68°C for 30 s, and cooling at 4°C. The resulting GC-clamped amplicons (3 μL) were electrophoresed on 1.5% agarose gels, which were then stained with GelRed (Biotium, Hayward, CA, USA) and examined under UV light.

A DGGE analysis of the amplified genes was performed using the DCode Universal Mutation Detection System (Bio-Rad, Hercules, CA, USA) according to the instruction manual. Aliquots (10 μL) of the PCR products were loaded onto polyacrylamide gels. Regarding general bacteria and *Alphaproteobacteria*, polyacrylamide gels (8% [w/v]) containing a linear formamide/urea gradient ranging between 30% and 70% were used and run at 100 V and 60°C for 16 h. Polyacrylamide gels (7% [w/v]) containing a linear formamide/urea gradient ranging from 20% to 45% were used for fungi and were run at 50 V and 60°C for 20 h.

Gels were stained with the GelStar Nucleic acid stain (Lonza, Basel, Switzerland) for 30 min, and the bands were visualized with a blue light transilluminator. The selected DGGE bands were excised and incubated in 10 μL of autoclaved H_2_O at 4°C overnight, and the supernatant (1 μL) was used as a template for PCR with the primer set without a GC clamp.

### Cloning and sequencing

The PCR product was purified using MonoFas (GL Science, Tokyo, Japan), ligated into the pMD19-T Simple Vector (Takara Bio, Shiga, Japan) using a DNA Ligation Kit (Takara), and transformed into competent *Escherichia coli* DH5α cells (Takara). Ten clones in each band were selected and colony PCR was performed using the primers M13 (5′-GTTTTCCCAGTCACGACGTT-3′) and M13R (5′-GGAAACAGCTATGACCATGA-3′) to amplify the insert. PCR products were treated with ExoSAP-IT (Affymetrix, Santa Clara, CA, USA), and both ends of DNA were sequenced using a BigDye Terminator v3.1 Kit and ABI 3130xl capillary sequencers (Applied Biosystems, Foster City, CA, USA).

### BPP sequence

The BPP-specific primers BPP-F and BPP-R (15) were used to amplify phytase gene fragments directly from metagenomic DNA or isolated IHP-utilizing bacteria with touchdown PCR. The reaction mixture (20 μL) contained 1× MightyAmp Buffer Ver. 2 (Takara), 1 μM of each primer, 0.5 U of MightyAmp DNA polymerase (Takara), and template DNA (*ca.* 20 ng) or 1 μL of the bacterial suspension. During touchdown PCR, the annealing temperature, which was initially set to 57°C, was decreased by 1°C every cycle until a touchdown of 48°C, at which temperature 27 additional cycles were performed. The PCR program was as follows: an initial denaturing step at 98°C for 2 min followed by 35 cycles of denaturation at 98°C for 10 s, 30 s for primer annealing at the appropriate temperature, extension at 68°C for 30 s, and cooling at 4°C. The size of the amplicon was measured by agarose gel electrophoresis and amplified fragments ranging between 160 and 200 bp in length were purified using MonoFas, ligated into the pMD19-T Simple Vector, and transformed into competent *E. coli* DH5α cells.

Based on a BLASTx analysis, the obtained DNA sequences that showed the highest level of identity to the validly published BPP protein sequences in the GenBank database were used as successfully amplified BPP gene fragments.

### Phylogenetic analysis

The 16S rRNA gene fragments and BPP gene fragments obtained were assembled as operational taxonomic units (OTUs) with similarities of greater than 95% and representative sequences were selected by the program mothur (29). The phylogenetic trees of the nucleotide sequences of the 16S rRNA gene or deduced amino acid sequences of BPP were constructed with MEGA version 5.0 (32) using the neighbor-joining method (27). The nucleotide sequences of BPP gene fragments were translated into amino acid sequences by EMBOSS Transeq (http://www.ebi.ac.uk/emboss/transeq) and aligned at the protein level using ClustalX (http://www.clustal.org/).

### Isolation and identification of IHP-utilizing bacteria

Four types of phytate screening media (PSM) (18) with some modifications were used to isolate and culture bacteria that utilize Na-IHP as the only phosphorous source. These media contained (L^−1^) 10 g or 0.1 g D-glucose, 4 g Na-phytate, 2 g CaCl_2_, 5 g NH_4_NO_3_, 0.5 g KCl, 0.5 g MgSO_4_·7H_2_O, 0.01 g FeSO_4_·7H_2_O, and 0.01 g MnSO_4_·H_2_O. The pH of the medium was adjusted to 7.0 with NaOH, and gelling agents were 1.5% agar or 1.5% gellan gum because some bacteria are culturable on gellan gum plates, but not on agar plates (10, 31). PSM with agar/high glucose (AH), agar/low glucose (AL), gellan gum/high glucose (GH), and gellan gum/low glucose (GL) were prepared.

A dilution method was employed to isolate IHP-utilizing strains from Ca-IHP or Fe-IHP alginate beads in Experiment 2 using four types of PSM plates. Alginate beads from five replicate pots were compiled and a 100-fold excess (v/w) of sterilized 0.85% NaCl solution was added and mixed well for 30 min at 200 rpm. Three replicates of dilution series were prepared and spread on each plate. The plates were incubated at 25°C in the dark. After 1 week, approximately 8 colonies from each plate type were removed and transferred to fresh plates repeatedly to obtain pure colonies.

In order to identify isolated strains, 16S rRNA sequence-based identification was performed using modified colony PCR. One loop of a colony was suspended in 50 μL of autoclaved distilled water, and 1 μL of the suspension was then added to 10 μL of the PCR mixture. 16S rRNA gene regions were amplified with KOD Fx Neo and sequenced using a direct PCR method. The first amplification of the 16S rRNA gene region used universal forward (27f) and reverse (1492r) primers (19). The PCR conditions used were as follows: preheating at 94°C for 2 min and 30 cycles of denaturation at 98°C for 10 s, annealing at 55°C for 30 s, and extension at 68°C for 90 s. In a direct PCR sequence analysis, an ABI Prism 3130xl genetic analyzer was used with a BigDye Terminator version 3.1 Cycle Sequencing Ready-Reaction Kit (Applied Biosystems).

### Degradation of Ca-IHP by IHP-utilizing bacteria

The Ca-IHP-degrading activity of IHP-utilizing bacteria was estimated by released inorganic P in limiting phytate-specific broth (26), which contained 1 mM Ca-IHP as the sole P source and (L^−1^) 1.0 g (NH_4_)_2_SO_4_, 0.1 g MgSO_4_·7H_2_O, 7.0 g KCl, 0.1 g CaCl_2_·2H_2_O, 1.0 mL of 0.1 M FeNa-EDTA, 1.0 mL of a complete trace element solution (L^−1^: 15.0 g Na_2_EDTA·2H_2_O, 0.43 g ZnSO_4_·7H_2_O, 0.24 g CoCl_2_·6H_2_O, 0.99 g MnCl_2_·4H_2_O, 0.22 g Na_2_MoO_4_·2H_2_O, 0.19 g NiCl_2_·6H_2_O, 0.08 g Na_2_SeO_3_·6H_2_O, and 0.15 g H_3_BO_4_). Test strains were grown in 5 mL of limiting phytate-specific broth with 1 mM Na-IHP shaken at 150 rpm at 25°C for 3 day. Cells were pelleted by centrifugation (3,500×*g*, 5 min), washed twice, resuspended in 0.85% NaCl solution, and adjusted to an OD_600_ of 0.1. Fifty microliters of the suspension was further cultivated in 10 mL limiting phytate-specific broth with horizontal shaking (150 rpm) at 25°C for 1 week. Released inorganic P was calculated from the change in the inorganic P concentration in the broth during the cultivation period; the concentration of inorganic P was measured by the malachite green method (5).

### Statistical analysis

All statistical analyses were performed using software R (https://www.r-project.org/). *P* values <0.05 were considered significant.

### Accession numbers

All gene sequences obtained in this study have been deposited in the DDBJ/EMBL/GenBank databases. Accession numbers LC101630 to LC101635, LC101620 to LC101627, LC101628 to LC101629, and LC101636 to LC101647 have been assigned to the BPP gene fragments cloned from OM, Ca-IHP beads, Fe-IHP beads, and 16 IHP-utilizing bacteria, respectively. Accession numbers LC101612 to LC101619 have been assigned to representative sequences obtained by DGGE. The accession numbers of the 16S rRNA gene sequences for the 28 strains isolated are LC101648 to LC101675.

## Results

### Shoot P content in Experiment 1

**Fig. 2:**
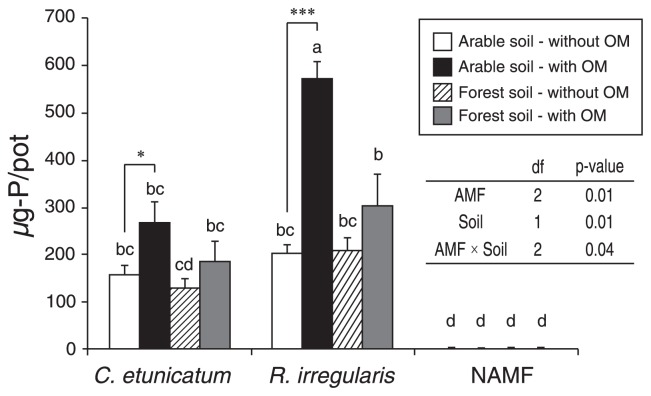
Shoot P contents of *L. japonicas* 6 weeks after transplanting under combinations of AMF, soil inoculums, and OM treatments (Experiment 1). Different letters indicate significant differences as assessed by Tukey’s HSD test (*P* <0.05). Asterisks indicate significant differences between the treatment with and without OM as assessed by the *t*-test (*P<0.05. ***P<0.001). Bars represent the SE of the means (*n*=4). The effects of AMF and the soil inoculum on the difference between with and without OM were analyzed using a two-way ANOVA for P contents. NAMF, no AMF.

Fig. 2 shows the shoot P content of host plants and Fig. S2 shows the shoot biomass of host plants. Shoot P contents were higher with the OM treatment than with the control (without OM), regardless of the combination of AMF and the soil inoculum under the AMF-inoculated treatment (Fig. 2). Shoot P contents increased 2.5-fold with *R. irregularis*-arable soil (*P* <0.001) and 1.7-fold with *C. etunicatum*-arable soil (*P* <0.05). AMF, soil inoculum, and AMF × soil inoculum interaction factors had significant effects on differences between the with and without OM applications (*P*=0.04), with the greatest difference being observed with the *R. irregularis-*arable soil inoculation. In the shoot P contents of AMF-infected plants without the OM application, we did not observe any significant differences under each combination of AMF and soil inocula. Changes in the shoot biomass were same as those in the shoot P content (Fig S2). Host plants without the AM inoculation hardly grew.

### Shoot P content in Experiment 2

**Fig. 3:**
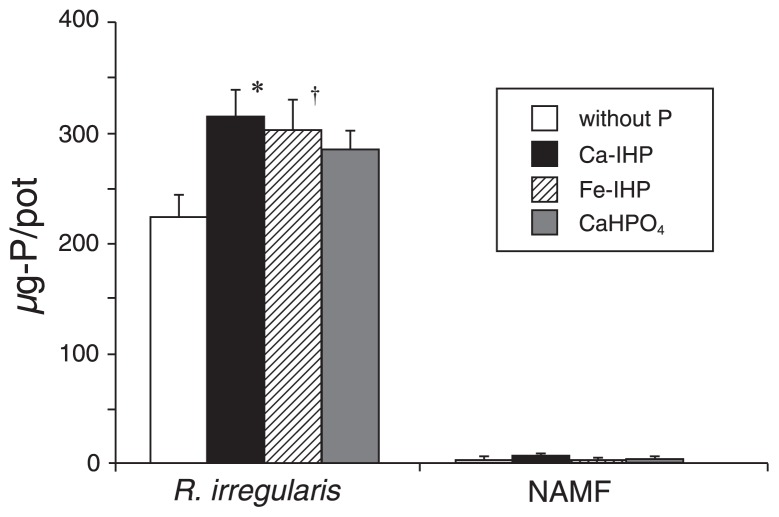
Shoot P contents of *L. japonicas* 6 weeks after transplanting with alginate beads containing each P source (Experiment 2). In each AMF treatment, the dagger and asterisk indicate significant differences between the control (without P) and treatments (Dunnett’s test; ^†^*P*<0.1, *P< 0.05). Bars represent SE of the means (*n*=5).

Fig. 3 shows the shoot P content of host plants and Fig. S3 shows the shoot biomass of host plants. In the AMF-inoculated treatment, the P contents of host plants were significantly higher with Ca-IHP beads (*P*=0.03) than with the control (P-free beads), and *P* values for the difference between the Fe-IHP bead and CaHPO_4_ bead treatments were 0.07 and 0.18, respectively. No significant differences were observed in the P contents of host plants between Ca-IHP, Fe-IHP, and CaHPO_4_ (Tukey’s HSD test). Changes in the shoot biomass were same as those in the shoot P content (Fig. S3). Host plants without the AM inoculation hardly grew. AMF spores were observed on Ca-IHP beads, and hyphae were observed on Ca-IHP beads and Fe-IHP beads (Fig. S4).

### DGGE analysis

**Fig. 4:**
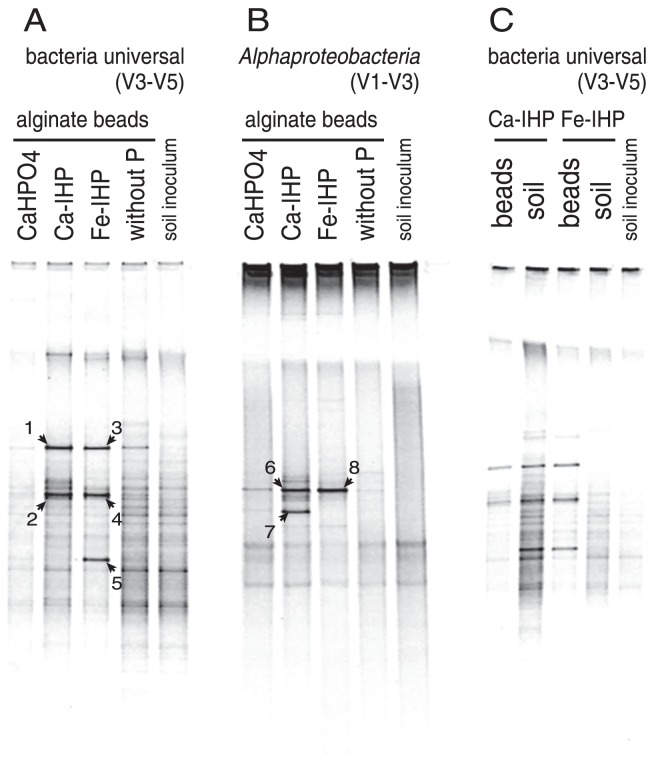
Denaturing gradient gel electrophoresis (DGGE) analysis based on 16S rRNA genes extracted from alginate beads and soil in the hyphal compartment of Experiment 2. Comparison of bacterial diversity nearby each P source in alginate beads using a bacterial universal primer (A) and *Alphaproteobacteria*-specific primer (B). Bacterial diversity around beads and soil using the bacterial universal primer (C). The numbered bands were cloned and sequenced.

DNA extracted from alginate beads and soils in Experiment 2 was analyzed by DGGE. The results of the DGGE analysis based on sequence differences in the V3–V5 region of the 16S rRNA genes targeting general bacteria are shown in Fig. 4A. The results of the DGGE analysis based on sequence differences in the V1–V3 region of the 16S rRNA genes targeting *Alphaproteobacteria* are shown in Fig. 4B. By DGGE targeting of the V3–V5 region (Fig. 4A), two pairs of bands were detected at the same position in the lanes of Ca-IHP beads and Fe-IHP beads (bands-1, −3 and bands-2, −4). The results of a BLAST search identified bands-1 and −3 as Burkholderiales. Bands-2 and −4 were identified as *Asticcacaulis* sp., *Caulobacter* sp., or *Sphingomonas* sp. with the same score. Band-5, which was specific to Fe-IHP, was identified as *Arthrobacter* sp. (Table 1). In the DGGE analysis targeting the V1–V3 region of *Alphaproteobacteria* (Fig. 4B), bands-6 and −8 were detected in the same position in the lanes of Ca-IHP beads and Fe-IHP beads. The results of the BLAST search identified bands-6 and −8 as *Sphingomonas* sp. (Table 1). Band-7 was detected as a specific band in the lanes of Ca-IHP beads and was identified as *Caulobacter* sp. (Table 1). The DGGE patterns targeting the general 16S rRNA of alginate beads containing IHP and soil surrounding alginate beads in mesh bags are shown in Fig. 4C, and only a few clear bands were detected in the lane of IHP-containing alginate beads. The DGGE band pattern targeting fungi was not different among alginate beads containing each type of P (Ca-IHP, Fe-IHP, CaHPO_4_, or no addition) and soil in the mesh bag (data not shown).

### Characteristics of the BPP gene

**Fig. 5:**
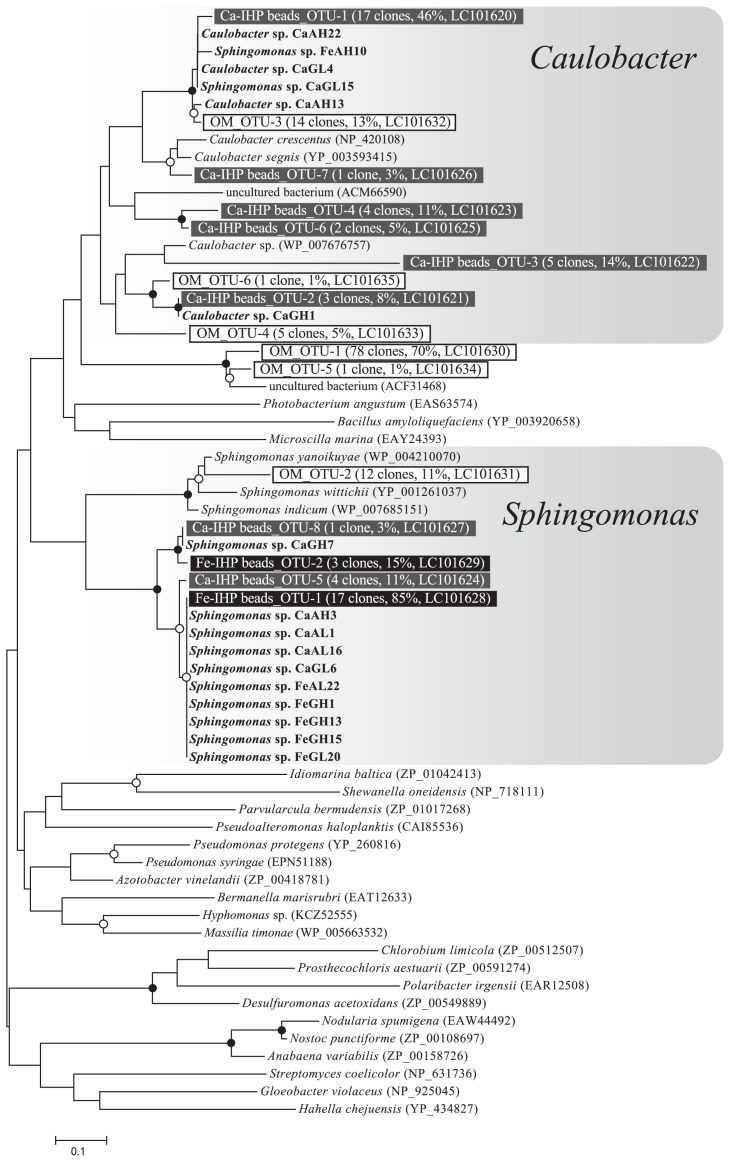
Phylogenetic trees showing the relationships between BPP amino acid sequences cloned from metagenomic DNA (OM from Exp. 1; Ca-, Fe-IHP alginate beads from Exp. 2) and isolated IHP-utilizing bacteria. The representative clones in each operational taxonomic unit (OTU) obtained from OM and Ca-, Fe-IHP alginate beads are indicated in white boxes or outlined characters. The numbers of clones obtained as the same OTU, relativity of colonies in the same sources, and accession number are shown in parentheses. The database accession numbers of isolated strains are shown in Table S1. Branch points supported with bootstrap values of >80% are marked with solid circles, while those supported with values of >50% are marked with open circles.

Amplified BPP-like gene fragments ranging between 160 and 200 bp in length were obtained from the extracted DNA of OM (*R. irregularis*-arable soil) in Experiment 1, Ca-IHP beads and Fe-IHP beads in Experiment 2, and some of the isolated IHP-utilizing bacteria. The numbers of BPP-like gene clones from OM, Ca-IHP beads, and Fe-IHP beads were 112, 37, and 20, respectively. Each clone was separated into a total of 16 OTUs based on a more than 95% DNA sequence similarity cut-off and translated to amino acid sequences. The tree topology of the BPP-like amino acid sequences showed two major clusters of *Sphingomonas* spp. and *Caulobacter* spp. (Fig. 5). The BPP-like gene clones amplified from OM were classified into the *Caulobacter* cluster (OM_OTU-3, −4, and −6; subtotal 18%), *Sphingomonas* cluster (OM_OTU-2; subtotal 11%), and an unidentified cluster (OM_OTU-1 and −5; subtotal 71%). The clones from Ca-IHP beads were classified into *Caulobacter* (Ca-IHP_OTU-1, −2, −3, −4, −6 and −7; subtotal 87%) and *Sphingomonas* clusters (Ca-IHP_OTU-5 and −8; subtotal 14%). The clones from Fe-IHP beads all belonged to the *Sphingomonas* cluster (Fe-IHP_OTU-1 and −2).

### Isolation of IHP-utilizing bacteria

**Fig. 6:**
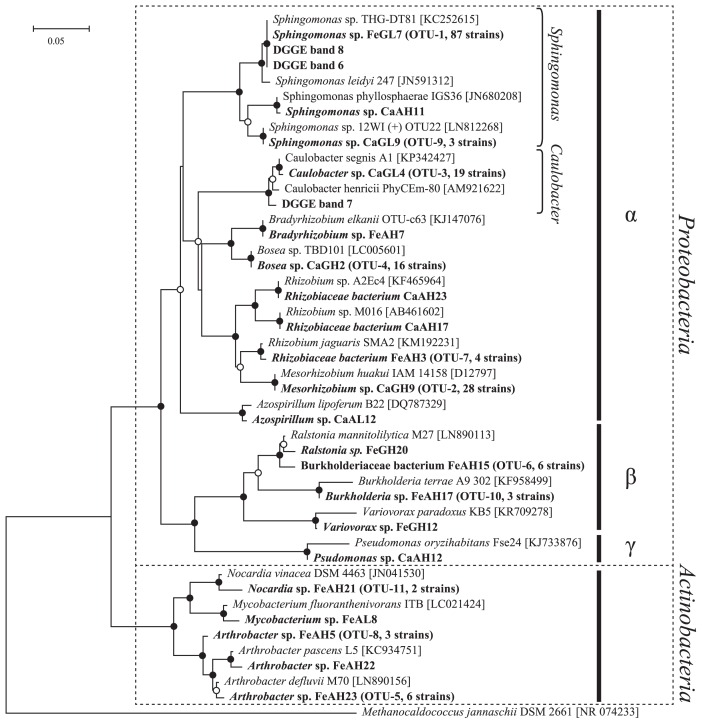
Phylogenetic analysis based on the 16S rRNA gene (V1–V3) of isolated IHP-utilizing bacteria and DGGE bands-6, −7, and −8. The representative strains in each operational taxonomic unit (OTU) are given in bold, and the numbers of isolates obtained as the same OTU are shown in parentheses. The database accession numbers of isolated strains are shown in Table S1. Branch points supported with bootstrap values of >80% are marked with solid circles, while those supported with values of >50% are marked with open circles.

Table 2 shows the number of IHP-utilizing bacteria isolated from alginate beads containing Ca-IHP and Fe-IHP recovered from the AMF-inoculated treatment in Experiment 2. A phylogenetic tree using a partial 16S rRNA gene (V1–V3) for the isolates and DGGE bands from Fig. 4B is shown in Fig. 6, and a phylogenetic tree using a partial 16S rRNA gene (V3) of the same strains and DGGE bands from Fig. 4A and Fig. 4B is shown in Fig. S5. *Burkholderiaceae* isolates (*Burkholderia* sp., *Burkholderiaceae* bacterium, *Ralstonia* sp., and *Variovorax* sp.) and the DGGE band from Fig. 4A (bands-1 and −3) were separated into different clusters (Fig. S5).

A total of 96 and 93 strains of IHP-utilizing bacteria were isolated from Ca-IHP beads and Fe-IHP beads, respectively. Most of the isolates were *Alphaproteobacteria* (Ca-IHP beads, 98%; Fe-IHP beads, 75%). Strains of *Sphingomonas* spp. were isolated from Ca-IHP beads and Fe-IHP beads, while strains of *Caulobacter* spp. were isolated from Ca-IP beads only (Table 2). The sequences of almost all isolated *Sphingomonas* spp., 87 strains in a subtotal of 91 strains, and *Caulobacter* spp., 14 strains in a subtotal of 19, were nearly identical. The phylogenetic positions of the BPP genes of these isolates were also included in Fig. 5, indicating that all BPP genes from the isolates were *Caulobacter* or *Sphingomonas*. The BPP gene of some isolates, *Sphingomonas* sp. CaGL15 and *Sphingomonas* sp. FeAH10, belonged to the *Caulobacter* cluster. The ratios of isolated proteobacteria strains were almost unchanged by the types of media (Table 2). On the other hand, *Actinobacteria* (*Arthrobacter* spp., *Mycobacterium* sp., and *Nocardia* spp.) were specific for agar media (AH and AL), even though previous studies showed the advantage of gellan gum for the isolation and incubation of *Actinobacteria* (10).

### Degradation of Ca-IHP by isolated IHP-utilizing bacteria

Table 3 shows inorganic P released from Ca-IHP, bacterial growth, and pH after an incubation in limiting phytate-specific broth, which contained Ca-IHP as the sole P source. Inorganic P increased significantly with *Sphingomonas* spp. CaAL16, CaGL4, FeGH1, FeGH15, *Caulobacter* sp. CaGL4, and *Variovorax* sp. FeGH12. Bacterial growth was observed in all strains, and a decrease in pH was observed in almost all strains, except *Burkholderiaceae* FeAH15. The BPP gene was not detected from *Actinobacteria* or *Burkholderia*/*Burkholderiaceae* isolates.

## Discussion

### P transfer from IHP to plants via AMF with IHP-degrading bacteria

P transfer from IHP to host plants via AMF has been reported in previous studies (7, 39); however, the IHP-degrading bacteria contributing to this phenomenon have not yet been isolated. In the present study, we confirmed the transfer of P from IHP via AMF and successfully isolated the dominant IHP-degrading bacteria from IHP in an AM fungal hyphal compartment using the baiting method.

In Experiment 1, *R. irregularis* and an arable soil were selected as an appropriate combination of AMF and an inoculum, which showed effective P transfer from IHP-rich OM to the plant (Fig. 2). In this experiment, a mixture of soybeans and buckwheat seeds was used as IHP-rich OM. Although IHP accounted for more than 60% of total P in OM, we cannot exclude the possibility of P transfer from sources other than IHP in OM to the plant. Therefore, alginate beads containing IHP were used instead of OM in Experiment 2.

In Experiment 2, evidence for P uptake from Ca-IHP and Fe-IHP via AMF was provided by increased P contents in host plants under the treatment with IHP beads than those under the P-free bead treatment (Fig. 3). Since AMF cannot hydrolyze IHP by themselves (35, 38), Ca-IHP- and Fe-IHP-degrading microbes appear to contribute to plant P uptake from IHP via AMF. Furthermore, similar levels of P uptake from Ca-IHP, Fe-IHP, and CaHPO_4_ suggest that IHP in OM is one of the important P sources for P uptake via AM fungi as well as inorganic P. It is important to note that applied IHP and inorganic P were adjusted to the amount of P in 0.5 g of soybeans.

### Microbial diversity in alginate beads containing IHP, evaluated with 16S rRNA gene and BPP gene sequences

DGGE profiles indicated that the bacterial flora in alginate beads containing IHP were simpler than those of soil (Fig. 4C). This result suggests that some specific groups of bacteria dominated by utilizing IHP in the beads. The active colonization of AM fungal extraradical hyphae and spore formation on Ca-IHP beads were observed by microscopy (Fig. S4). This result further suggests that the dominant bacteria in IHP beads, as shown in the DGGE profiles, may contribute to P release from IHP in the beads and that the P released may be absorbed by AMF and transferred to plants.

The DGGE profiles of Ca-IHP beads based on the V3–V5 region of the bacterial 16S rRNA gene showed two major bands (Fig. 4A): the sequence of band-1 was identified as *Burkholderiales* and the sequence of band-2 overlapped with three *Alphaproteobacteria* (*Asticcacaulis* sp., *Caulobacter* sp., and *Sphingomonas* sp.). On the other hand, the analysis of BPP genes around Ca-IHP beads suggests that dominant IHP-degrading bacteria belonged to two clusters of *Alphaproteobacteria*, *Sphingomonas*, and *Caulobacter* (Fig. 5). Therefore, in order to elucidate the origin of band-2, a further DGGE analysis targeting another region, V1–V3, of *Alphaproteobacteria* was performed, and bands for *Sphingomonas* sp. and *Caulobacter* sp. (bands-6 and −7) were detected. The BPP-like genes amplified from Ca-IHP beads also belonged to *Sphingomonas* sp. and *Caulobacter* sp. Based on these results, the dominant IHP-degrading bacteria around Ca-IHP appear to be *Sphingomonas* sp. and *Caulobacter* sp. It is important to note that *Sphingomonas* spp. and *Caulobacter* spp. were the major strains of IHP-utilizing bacteria isolated from Ca-IHP beads (Table 2).

The DGGE profiles of Fe-IHP beads based on the V3–V5 region of the bacterial 16S rRNA gene showed three major bands (Fig. 4A): the sequence of band-3 was identified as *Burkholderiales* and the sequence of band-4 overlapped with three *Alphaproteobacteria* (*Asticcacaulis* sp., *Caulobacter* sp., and *Sphingomonas* sp.), and band-5 was identified as *Arthrobacter* sp. On the other hand, the results of the analysis of BPP genes around Fe-IHP beads suggest that dominant IHP-degrading bacteria belonged to the *Sphingomonas* cluster (Fig. 5). The DGGE analysis targeting the V1–V3 region of *Alphaproteobacteria* showed only one band (band-8) for *Sphingomonas* sp. (Fig. 4B). These results suggest that the dominant IHP-degrading bacteria around Fe-IHP were *Sphingomonas* sp. Similar to Ca-IHP, most IHP-utilizing bacteria isolated from Fe-IHP beads may be dominant members of the microbial community on Fe-IHP beads.

Therefore, the baiting method using alginate beads containing IHP was successfully adapted to our research. Isolated *Sphingomonas* spp. are considered to be one of the dominant bacteria on alginate beads because the sequences of *Sphingomonas* spp. (band-6, −8, Fig. 4B, Table 1) were nearly identical to the major bands of Ca-IHP beads and Fe-IHP beads (Fig. 6, Table S1), and the BPP of isolated IHP-utilizing bacteria (including FeAL22 and FeGH1) was nearly identical to the major OTU of Fe-IHP beads, Fe-IHP_OTU-1 (Fig. 5).

Various soil fungi exhibit phytase activity. Tarafdar and Marschner (34) reported enhancements in plant P uptake from IHP by fungi. However, IHP-utilizing fungi were not considered in the present study because the DGGE profiles of fungi were not affected by the P source in alginate beads (Ca-IHP, Fe-IHP, and inorganic P, data not shown). Therefore, we focused on IHP-degrading bacteria. Further studies are needed in order to elucidate how soil fungi are involved in AMF-mediated P transfer from IHP to plants.

### IHP degradation by IHP-utilizing isolates

The accumulation of inorganic P in Ca-IHP medium by the incubation of isolated IHP-utilizing strains shows that four *Sphingomonas* strains (CaAL16, CaGL15, FeGH1, and FeGH15), a *Caulobacter* strain (CaGL1), and *Variovorax* strain (FeGH12) were IHP-degrading bacteria (Table 3). In addition, the BPP gene was amplified from all of these strains, except for the *Variovorax* strain. Ca-IHP is dissolved in medium by reducing pH (16); however, the compound is not hydrolyzed under such pH conditions. Acidic conditions may be caused by acidic compounds released from bacteria and may facilitate the hydrolysis of IHP by bacterial phytase (8). The other 6 strains among the 12 strains did not release inorganic P from Ca-IHP. However, this test used monocultures in liquid medium, which may be influenced by other microbes, whereas solid experimental conditions more closely resemble the soil environment. Not only *Sphingomonas* spp. and *Caulobacter* spp., but also *Arthrobacter* spp. and *Burkholderiaceae* may degrade IHP under soil conditions because the degradation of IHP by these bacteria has been reported previously (12, 37). However, Fig. S5 suggests that *Burkholderiaceae* isolates were not dominant on alginate beads in Experiment 2.

To the best of our knowledge, this is the first study to show the IHP-degrading activities of soil bacteria belonging to *Sphingomonas* and *Caulobacter*. These bacteria are ubiquitous in the environment (*e.g.*, soil, rhizosphere, and aquatic systems), and BPP genes have been detected in their genomes (20). The distribution of IHP-utilizing *Caulobacter* spp. and *Sphingomonas* spp. around OM in Experiment 1 was suggested because BPP clones amplified from OM belonged to the *Caulobacter* and *Sphingomonas* clusters, even though the ratio was not very high (Fig. 5). IHP-utilizing bacteria carrying dominant BPP (OM_OTU_1) were not isolated. This result indicates that IHP-utilizing *Caulobacter* and *Sphingomonas* are involved in the degradation of IHP in plant residues in soil. Further investigations on the role of these bacterial groups in IHP dynamics in soil are needed.

### Interactions between AMF and soil microbes

In Experiment 1, plant P uptake in the treatment without OM was similar regardless of the combination of AMF and the soil inoculum (Fig. 2). However, a significant increase in P uptake was only found with the combination of *R. irregularis* and the arable soil inoculum, suggesting that this specific combination may facilitate P transfer from OM to plants through the degradation of organic P in IHP-rich OM by soil microbes derived from arable soil. This raises the hypothesis that the combination of AMF and microbes may be crucial in the dynamics of organic P in soil. Some soil bacterial activities such as OM decomposition, N mineralization, and IHP degradation have been shown to be accelerated by AMF (4, 11, 39). However, the specific combination of AMF and soil microbes was not considered in these studies. Since the influence of AMF on bacterial activity may vary according to the particular combination of AMF and soil microbes, it will likely be important to elucidate the mechanisms of P transfer from IHP to plants via AMF.

## Conclusion

P transfer from IHP via AMF with the assistance of IHP-degrading bacteria was confirmed. Using a modified baiting method with alginate beads, *Sphingomonas* spp. and *Caulobacter* sp. were isolated as IHP-degrading bacteria from the IHP in the hyphal compartment of AMF. Their predominance on beads containing IHP suggests their contribution to P transport from IHP via AMF. In order to demonstrate the involvement of these IHP-degrading bacteria in AMF-mediated P transfer from IHP to plants, further experiments such as dual inoculation tests using AMF and these isolates are required. Ca-IHP is dissolved under acidic conditions (16); therefore, microbes may solubilize Ca-IHP in soil through the release of organic acids (8). However, the degradation of Fe-IHP by microbes has not yet been observed because Fe-IHP is hardly dissolved under acidic conditions (16). Further studies on the degradation of Fe-IHP by IHP-utilizing bacteria isolated from Fe-IHP-containing alginate beads are in progress and will be reported elsewhere.

## Supplementary Information



## Figures and Tables

**Table 1:** Taxa, closest relatives as determined by sequence identity, and accession numbers for bands on DGGE targeting 16S rRNA

band No.^a^	Closest relative (obtained by a BLAST sesarch)	Identity (%)	seq region	Accession no.
1	*Burkholderiales*	98.5	V3–V5	LC101612
2	*Sphingomonas* sp., *Caulobacter* sp., *Asticcacaulis* sp.^b^	99.8	V3–V5	LC101613
3	*Burkholderiales*	98.8	V3–V5	LC101614
4	*Sphingomonas* sp., *Caulobacter* sp., *Asticcacaulis* sp.^b^	99.8	V3–V5	LC101615
5	*Arthrobacter* sp.	99.8	V3–V5	LC101616
6	*Sphingomonas* sp. THG-DT81	100.0	V1–V3	LC101617
7	*Caulobacter* sp. HDXJ05	99.4	V1–V3	LC101618
8	*Sphingomonas* sp. THG-DT81	100.0	V1–V3	LC101619

a Bands shown in Fig. 4A and 4B.

b Multiple relatives were given in the same score.

**Table 2:** Taxonomic identification of 187 isolated IHP-utilizing strains based on 16S rRNA sequences

	No. of strains (AH, AL, GH, GL)^a^	
	
	Ca-IHP^b^	Fe-IHP^b^
*Alphaproteobacteria*		
*Azospirillum* sp.	1 (0, 1, 0, 0)	0
*Bosea* spp.	14 (1, 4, 7, 2)	2 (0, 1, 1, 0)
*Bradyrhizobium* sp.	0	1 (1, 0, 0, 0)
*Caulobacter* spp.	19 (5, 6, 3, 5)	0
*Mesorhizobium* spp.	22 (7, 3, 5, 7)	6 (1, 1, 3, 1)
*Rhizobiaceae* bacterium	4 (4, 0, 0, 0)	2 (2, 0, 0, 0)
*Sphingomonas* spp.	34 (5, 10, 9, 10)	57 (3, 16, 17, 21)
*Betaproteobacteria*		
*Burkholderia* spp.	0	3 (2, 0, 1, 0)
*Burkholderiaceae* bacterium	0	6 (5, 1, 0, 0)
*Ralstonia* sp.	0	1 (0, 0, 1, 0)
*Variovorax* sp.	0	1 (0, 0, 1, 0)
*Gammaproteobacteria*		
*Pseudomonas* sp.	1 (1, 0, 0, 0)	0
*Actinobacteria*		
*Arthrobacter* sp.	1 (1, 0, 0, 0)	9 (7, 2, 0, 0)
*Mycobacterium* sp.	0	1 (0, 1, 0, 0)
*Nocardia* sp.	0	2 (1, 1, 0, 0)
total	96	91

a The numbers in parentheses are the numbers of isolated strains using AH, AL, GH, and GL; AH, phyte screening media with agar/high glucose; AL, agar/low glucose; GH, gellan gum/high glucose; GL, gellan gum/low glucose.

b Isolation source; Ca and Fe indicate Ca-IHP containing alginate beads and Fe-IHP containing alginate beads.

**Table 3:** Characterization of IHP-utilizing bacteria isolated from Ca-IHP and Fe-IHP beads from Experiment 2

strain name	OTU in Fig. 6	closest relative	identity	BPP^a^	source^b^	P release (μmol mL^−1^)^c^	Biomass (OD_600_)^d^	pH^e^
*Sphingomonas* sp. CaAL16	OTU-1	*Sphingomonas* sp. THG-DT81	100%	+	Ca-IHP	1.83 ± 0.11 ***	2.00 ± 0.10	3.6 ± 0.0 ***
*Sphingomonas* sp. CaGL15	OTU-9	*Sphingomonas* sp. GB1	99%	+	Ca-IHP	0.59 ± 0.03 ***	1.71 ± 0.13	4.2 ± 0.0 ***
*Sphingomonas* sp. FeGH1	OTU-1	*Sphingomonas* sp. THG-DT81	100%	+	Fe-IHP	2.56 ± 0.09 ***	1.91 ± 0.10	3.9 ± 0.1 ***
*Sphingomonas* sp. FeGH15	OTU-1	*Sphingomonas* sp. THG-DT81	100%	+	Fe-IHP	0.52 ± 0.03 ***	1.80 ± 0.04	4.0 ± 0.0 ***
*Caulobacter* sp. CaGH1	OTU-3	*Caulobacter* sp. BBCT11	100%	+	Ca-IHP	0.27 ± 0.00	1.65 ± 0.06	4.3 ± 0.1 ***
*Caulobacter* sp. CaGL4	OTU-3	*Caulobacter* sp. ptl1	100%	+	Ca-IHP	0.43 ± 0.00 **	1.42 ± 0.04	4.0 ± 0.0 ***
*Arthrobacter* sp. FeAH9	OTU-5	*Arthrobacter* sp.	100%	−	Fe-IHP	0.23 ± 0.01	1.94 ± 0.11	3.8 ± 0.1 ***
*Arthrobacter* sp. FeAL9	OTU-8	*Arthrobacter* sp.	100%	−	Fe-IHP	0.19 ± 0.00	0.60 ± 0.04	4.0 ± 0.0 ***
Control 1^f^						0.12 ± 0.00		7.1 ± 0.0
*Burkholderiaceae* FeAH15	OTU-6	*Burkholderiaceae* bacterium CRh1	96%	−	Fe-IHP	0.17 ± 0.00	0.13 ± 0.02	7.2 ± 0.0
*Burkholderia* sp. FeAH17	OTU-10	*Burkholderia terrae* A9 302	96%	−	Fe-IHP	0.18 ± 0.01	3.77 ± 0.04	4.7 ± 0.0 ***
*Ralstonia* sp. FeGH20		*Ralstonia* sp. RZ2MS21	95%	−	Fe-IHP	0.14 ± 0.00	3.62 ± 0.02	4.2 ± 0.0 ***
*Variovorax* sp. FeGH12		*Variovorax paradoxus* KB5	96%	−	Fe-IHP	0.21 ± 0.01 **	0.12 ± 0.04	4.9 ± 0.0 ***
Control 2^g^						0.16 ± 0.00		7.2 ± 0.0

a Amplification of BPP fragment; −, indicates no amplification; +, amplified.

The fragments successfully amplified were sequenced and analyzed in Fig. 5.

b Isolation source; Ca and Fe indicate Ca-IHP containing alginate beads and Fe-IHP containing alginate beads.

c Phosphate release from Ca-IHP during a 7-day cultivation period.

d OD_600_ increment over the cultivation period as a measure of biomass increase.

e pH of medium after the cultivation period.

c, d, e Data represent the mean±SE (*n*=3).

c, e Asterisk indicate a significant difference between the control (without an inoculation) and treatment (Dunnett’s test; ** *P*<0.01, ***P <0.001).

f Control 1 were incubated at the same time as *Sphingomonas* spp., *Caulobacter* spp., and *Arthrobacter* spp.

g Control 2 were incubated at the same time as *Burkholderiaceae*, *Burkholderia* sp., *Variovorax* sp., and *Ralstonia* sp.
