# Supplementary material for: Isolation of Inositol Hexaphosphate (IHP)-Degrading Bacteria from Arbuscular Mycorrhizal Fungal Hyphal Compartments Using a Modified Baiting Method Involving Alginate Beads Containing IHP
Source: Microbes Environ. 2016 Jul 5;31(3):234–43. doi: 10.1264/jsme2.ME15206 (PMC5017799; doi:10.1264/jsme2.ME15206)
Supplement: Supplementary file 1 [file 31_234_s1.pdf]

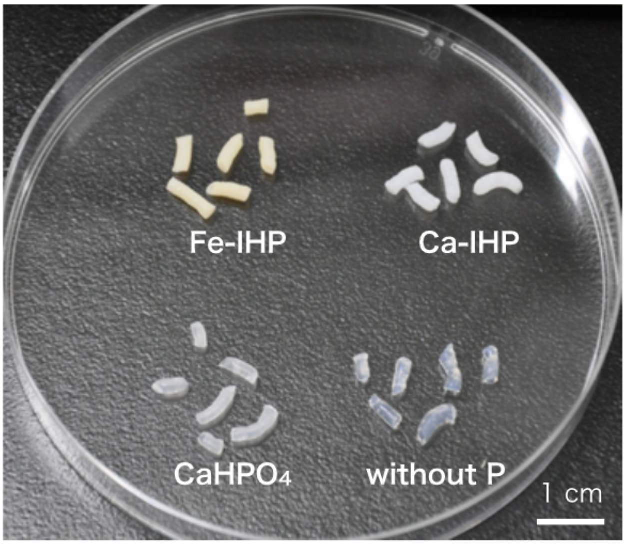

Fig. S1. Alginate beads containing each P source

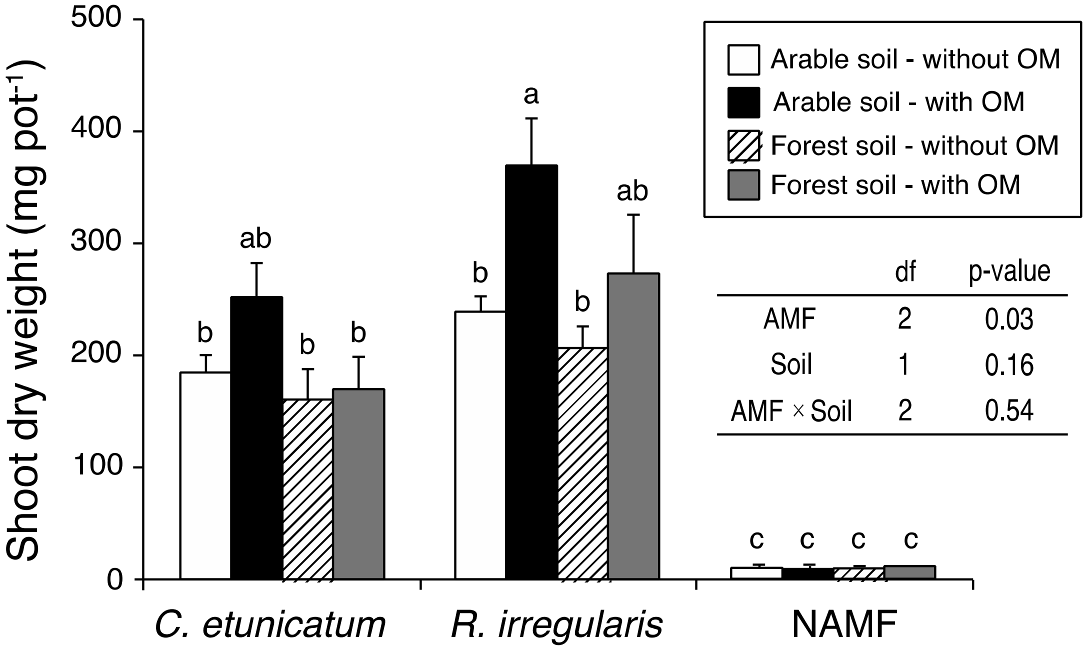

Fig. S2. Shoot dry weight of *L. japonicas* 6 weeks after transplanting under combinations of AMF, soil inoculums, and OM treatments (Experiment 1). Different letters indicate significant difference as assessed by Tukey's HSD test ($P < 0.05$). Bars represent SE of the means (n=4).
NAMF, no AMF

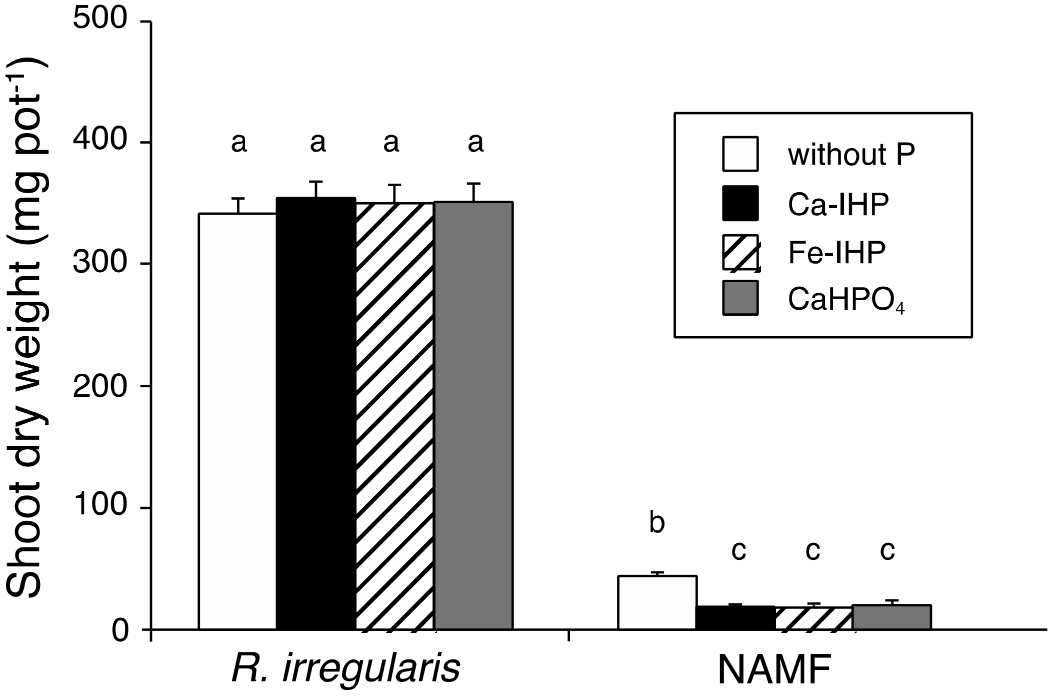

Fig. S3. Shoot dry weight of *L. japonicas* at 6 weeks after transplanting with alginate beads containing each P source (Experiment 2). Different letters indicate significant difference as assessed by Tukey's HSD test ($P < 0.05$). Bars represent SE of the means (n=5).
NAMF, no AMF

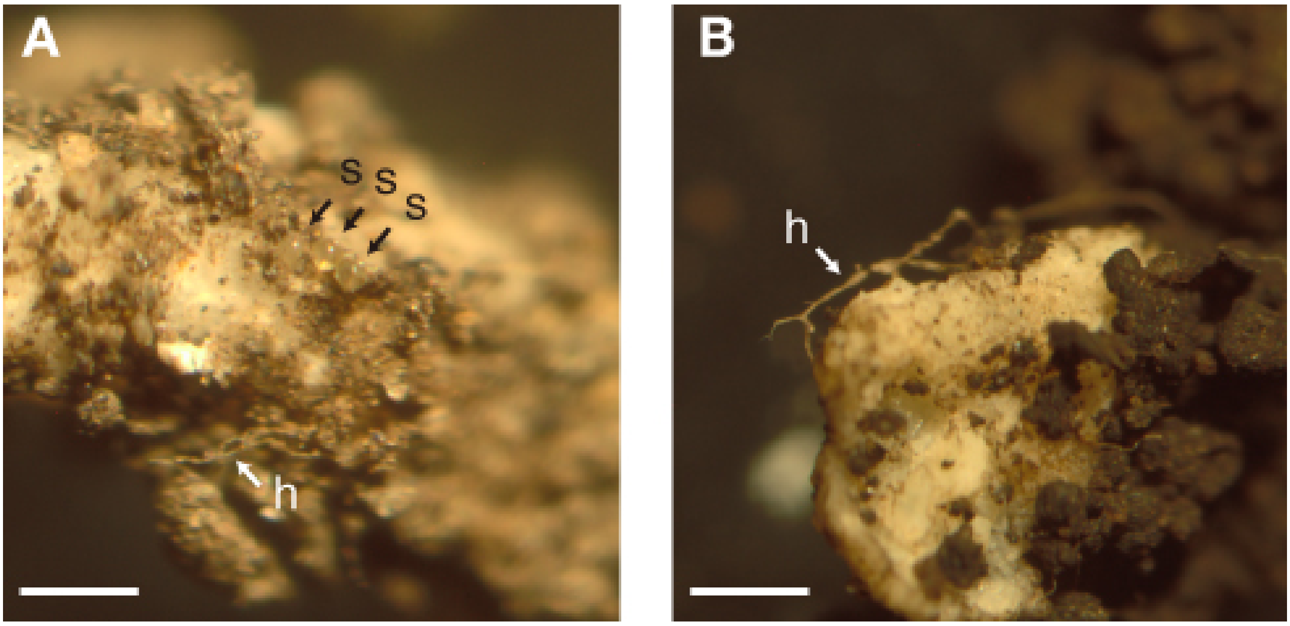

Fig. S4. AMF on alginate beads containing Ca-IHP (A) and Fe-IHP (B)

s, spore; h, hyphae. Bar=500 µm

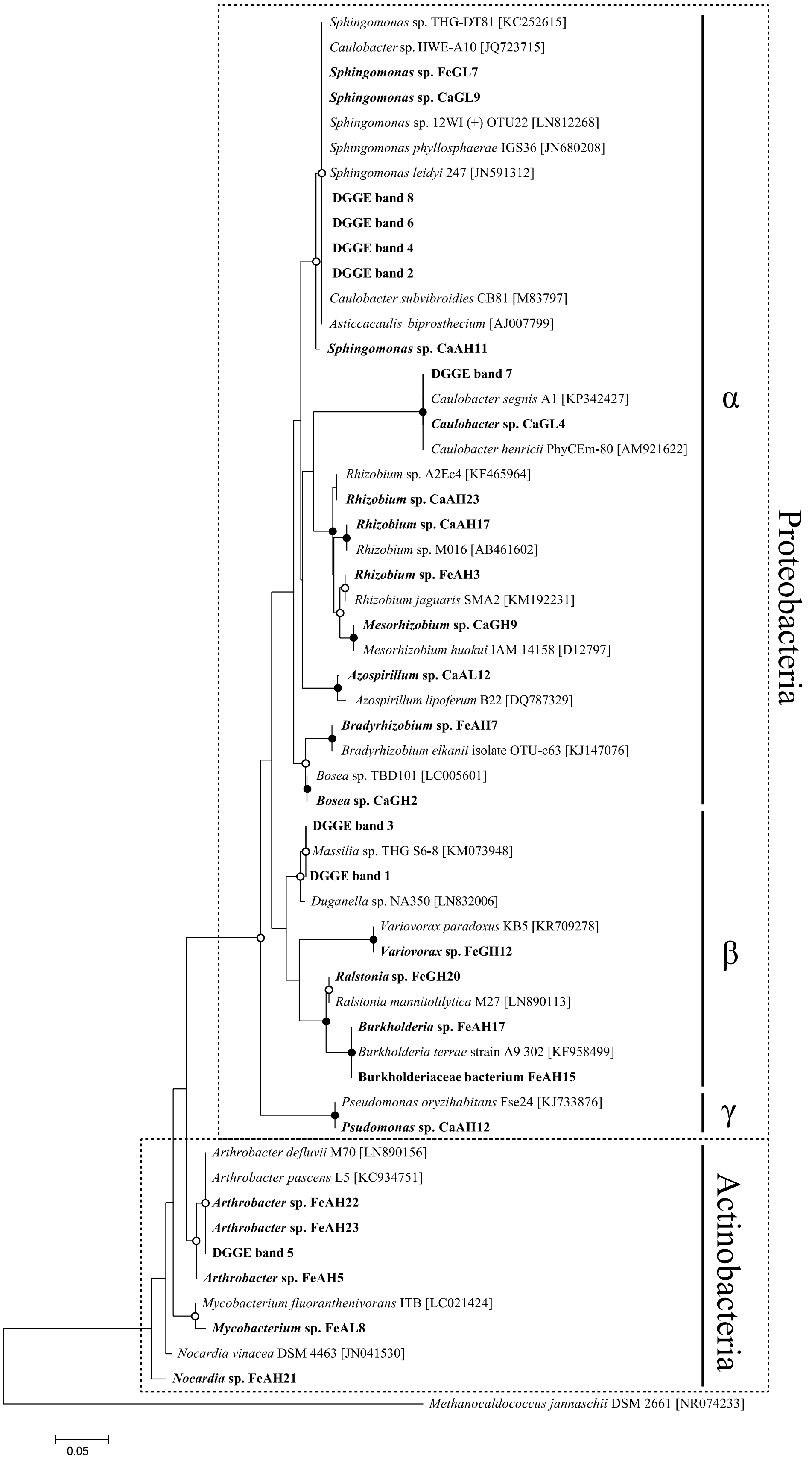

Fig. S5. Phylogenetic analysis based on 16S rRNA gene (V3) of isolated IHP-utilizing bacteria and DGGE band in Fig. 4A and Fig. 4B. The isolated IHP-utilizing bacteria used in Fig 6 are given in bold. Branch points supported with bootstrap values of >80% are marked with solid circles, while those supported with values of >50% are marked with open circles.

Table S1. Strains of each OTU in Fig. 6

Closest species	OTU in Fig. 6	Strain name[a] (accession numbers of BPP-like gene or 16S rRNA gene in parentheses)
Sphingomonas sp.	OTU-1	CaAH3 (BPP: LC101636), CaAH6, CaAH15, CaAH24, CaAL1 (BPP: LC101637), CaAL5, CaAL9, CaAL11, CaAL13, CaAL15, CaAL16 (16S: LC101652), CaAL17, CaAL23, CaAL24, CaGH3, CaGH7 (BPP: LC101643), CaGH8, CaGH10, CaGH13, CaGH16, CaGH18, CaGH22, CaGH23, CaGL1, CaGL2, CaGL3, CaGL6, CaGL10, CaGL16, CaGL19, CaGL24, FeAH16, FeAH19, FeAL1, FeAL5, FeAL6, FeAL7, FeAL10, FeAL11, FeAL12, FeAL14, FeAL15, FeAL18, FeAL19, FeAL20, FeAL21, FeAL22 (BPP: LC101638), FeAL23, FeAL24, FeGH1 (16S: LC101654; BPP: LC101639), FeGH2, FeGH3, FeGH4, FeGH5, FeGH7, FeGH8, FeGH10, FeGH13 (BPP: LC101640), FeGH15 (16S: LC101655), FeGH16, FeGH18, FeGH19, FeGH21, FeGH22, FeGH23, FeGH24, FeGL1, FeGL2, FeGL3, FeGL4, FeGL6, **FeGL7 (16S: LC101674)**, FeGL8, FeGL10, FeGL11, FeGL12, FeGL13, FeGL14, FeGL16, FeGL17, FeGL18, FeGL19, FeGL20 (BPP: LC101641), FeGL21, FeGL22, FeGL23, FeGL24
Mesorhizobium sp.	OTU-2	CaAH1, CaAH7, CaAH9, CaAH10, CaAH16, CaAH18, CaAH19, CaAL4, CaAL19, CaAL22, CaGH4, **CaGH9 (16S: LC101663)**, CaGH11, CaGH14, CaGH17, CaGL5, CaGL11, CaGL12, CaGL14, CaGL18, CaGL21, CaGL23, FeAH6, FeAL13, FeGH6, FeGH14, FeGH17, FeGL5
Caulobacter sp.	OTU-3	CaAH2, CaAH13 (BPP: LC101644), CaAH2, CaAH21, CaAH22 (BPP: LC101645), CaAL7, CaAL10, CaAL14, CaAL2, CaAL20, CaAL21, CaGH1 (16S: LC101650 ; BPP: LC101647), CaGH21, CaGH24, **CaGL4 (16S: LC101651 ; BPP: LC101646)**, CaGL7, CaGL13, CaGL17, CaGL22
Bosea sp.	OTU-4	CaAH4, CaAL3, CaAL6, CaAL8, CaAL18, **CaGH2 (16S: LC101659)**, CaGH5, CaGH6, CaGH12, CaGH15, CaGH19, CaGH20, CaGL8, CaGL20, FeAL4, FeGL15
Arthrobacter sp.	OTU-5	CaAH8, FeAH14, **FeAH23 (16S: LC101656)**, FeAH24, FeAH9 (16S: LC101648), FeAL17
Burkholderiaceae	OTU-6	FeAH11, **FeAH15 (16S: LC101662)**, FeAH2, FeAH4, FeAH8, FeAL2
Rhizobium sp.	OTU-7	CaAH5, CaAH14, **FeAH3 (16S: LC101669)**, FeAH20
Arthrobacter sp.	OTU-8	FeAH18, **FeAH5 (16S: LC101657)**, FeAL9 (16S: LC101649)
Sphingomonas sp.	OTU-9	**CaGL9 (16S: LC101673)**, CaGL15 (16S: LC101653; BPP: LC101642), FeAH10
Burkholderia sp.	OTU-10	FeAH1, **FeAH17 (16S: LC101661)**, FeGH11
Nocardia sp.	OTU-11	**FeAH21 (16S: LC101666)**, FeAL16
Arthrobacter sp.		**FeAL8 (16S: LC101665)**
Azospirillum sp.		**CaAL12 (16S: LC101658)**
Bradyrhizobium sp.		**FeAH7 (16S: LC101660)**
Micrococcus sp.		**FeAH22 (16S: LC101664)**
Psudomonas sp.		**CaAH12 (16S: LC101667)**
Ralstonia sp.		**FeGH20 (16S: LC101668)**
Rhizobium sp.		**CaAH17 (16S: LC101670)**
Rhizobium sp.		**CaAH23 (16S: LC101671)**
Sphingomonas sp.		**CaAH11 (16S: LC101672)**
Variovorax sp.		**FeGH12 (16S: LC101675)**

[a]The first letter indicates the isolation source and the type of phytate screening media used for isolation; Ca stands for alginate beads containing Ca-IHP; Fe stands for alginate beads containing Fe-IHP; AH stands for agar/high glucose; AL stands for agar/low glucose; GH stands for gellan gum/high glucose; GL stands for gellan gum/low glucose. Bold font strains were shown in Fig. 6
